# Pilus-mediated co-aggregation with *Lactobacillus crispatus* increases meningococcal susceptibility to antimicrobial agents by interfering with microcolony formation

**DOI:** 10.1186/s12866-025-04201-2

**Published:** 2025-07-30

**Authors:** Kenny Lidberg, Sarah Pilheden, Samuddi Nawarathne, Katharina Rauscher, Ann-Beth Jonsson

**Affiliations:** Department of Molecular Biosciences, The Wenner-Gren Institute, Stockholm University, Stockholm, SE-10691 Sweden

**Keywords:** *Neisseria meningitidis*, *Lactobacillus*, Aggregation, LL-37, HBD2, Antibiotics

## Abstract

**Background:**

*Neisseria meningitidis* asymptomatically colonizes the nasopharyngeal mucosa, but occasionally, the bacteria disseminate to cause sepsis and meningitis. In the epithelial cell layer, the pathogen co-colonizes with other resident inhabitants, such as *Lactobacillus* species that are part of the nasopharyngeal-oral microbiota. In this study, we investigated the interaction between lactobacilli and meningococci.

**Results:**

We demonstrated that *Lactobacillus crispatus* strain MV24 can co-aggregate with meningococci, whereas all other *Lactobacillus* strains tested did not co-aggregate. The binding ability of *L. crispatus* was not strain- or serogroup-specific but was dependent on the ability of meningococci to form microcolonies. The finding that *N. meningitidis* lacking pili did not co-aggregate with *L. crispatus*, but that hyperpiliated *N. meningitidis* exhibited strong co-aggregation, led us to examine the interaction between purified meningococcal pili and lactobacilli. Our results showed that *L. crispatus* MV24 can bind to purified meningococcal Class I and II pili, explaining the aggregative clusters observed under the microscope. Co-aggregation with *L. crispatus* disrupted microcolony formation, and increased the killing of meningococci by LL-37, hBD2 and cephalexin. Further, co-aggregation had the added effect of impeding motility.

**Conclusion:**

*N. meningitidis* pili bind to *L. crispatus*, which interferes with the meningococcal microcolonies and increases sensitivity to antimicrobial agents. Taken together, our findings suggest that *L. crispatus* MV24 may have a beneficial effect on the host through co-aggregating with meningococci.

**Supplementary Information:**

The online version contains supplementary material available at 10.1186/s12866-025-04201-2.

## Introduction

The human nasopharyngeal mucosa, located between the nose and oropharynx, is colonized by a large variety of bacterial species that constitute the nasopharyngeal microbiota. This microbial community continuously changes in diversity due to, for example, the aging of the host, seasonal fluctuations, or upper respiratory infections that disrupt the local microenvironment [1, 2]. Among the residents of the nasopharyngeal mucosa is the gram-negative obligate human colonizer *Neisseria meningitidis* (meningococcus). Meningococcal asymptomatic carriage ranges from 5 to 35% and constitutes approximately 2% of the adult nasopharyngeal microbiome [2, 3]. For reasons not fully understood, meningococci occasionally cross the mucosal epithelial barrier, proliferate in the bloodstream to cause sepsis and cross the blood-brain barrier to cause meningitis [4, 5].

Transmission between carriers occurs primarily via aerosol droplets. Upon reaching the nasopharyngeal mucosa, meningococci express many virulence factors that promote survival and the colonization of host epithelial cells. Bacterial numbers are restricted within the mucosa with the help of antimicrobial proteins such as secretory IgA, lysozyme, and antimicrobial peptides, i.e., the cathelicidin LL-37 and the human defensins [6, 7]. Meningococci can overcome host immune innate defenses via the production of a polysaccharide capsule (divided into serogroups A, B, C, X, Y and W-135), the modification of LPS, the expression of IgA protease, and the inhibition of lysosome maturation [8–11].

At mucosal surfaces, Type IV pili (Tfp) play critical roles in motility, adherence to the host cells, and the formation of bacterial aggregates. Meningococcal Tfp are categorized into Class I and Class II pili, which differ in both structure and function, including their capacity to undergo antigenic variation and posttranslational modifications such as glycosylation (*pgl* genes) and phosphorylation by phosphoglycerol transferase A or B (PptA/PptB) [12, 13].

Meningococci can form spherical structures known as microcolonies, consisting of anywhere from a dozen to several thousand bacterial cells. The major subunit PilE, along with several minor subunits, is required for successful formation into a microcolony [14–16]. The ATPases PilF, which mediates pilus elongation via the secretin PilQ, and PilT, which enables pilus retraction, confer dynamic properties to the pili and are necessary for both the formation of microcolonies and their subsequent dispersal into single cells [15, 17]. Additionally, the upregulation of anti-aggregation factor A (NafA) is involved in bacterial aggregation and piliation [18].

The dynamics of pathogenic *Neisseria* microcolonies depend on the movement of individual cells within the structure and the strength of the Tfp-Tfp interactions. These factors collectively determine the shape, size, and behavior of the microcolony, which in turn influence its tolerance to antimicrobial substances. Notably, nonpiliated and nonaggregative PilE-mutants exhibit increased susceptibility to antibiotics and antimicrobial peptides [19–21].

The microbiota of the healthy human upper respiratory tract is complex. *Lactobacillus* spp have a niche in the human nasopharynx and are abundant after birth and early childhood, especially the vaginal strains *L. crispatus* and *L. gasseri* in children born naturally, but they are still present in adults to a lesser degree [2, 22]. Lactobacilli are gram-positive bacteria with a cell wall that contains an S-layer and associated positively charged proteins that are essential for binding to host cells and other bacteria [23, 24]. Lactobacilli support their host and possess a diverse arsenal of antipathogenic properties. They can directly kill pathogens by secreting antimicrobial substances such as hydrogen peroxide and lactic acid, which acidify the local milieu [25, 26]. Alternatively, they inhibit pathogens through competitive exclusion, outcompeting them for host receptor binding, or through co-aggregation, where lactobacilli trap pathogens in aggregates that are cleared by bodily fluids [27–30].

In this study, we investigated the interaction between lactobacilli and meningococci, which are both members of the nasopharyngeal microbiota. We showed that *L. crispatus* MV24, but not the other *Lactobacillus* strains tested, co-aggregated with meningococcal microcolonies. Furthermore, this *L. crispatus* strain bound to purified meningococcal pili. Co-aggregation with *L. crispatus* disrupted microcolony formation, leading to increased sensitivity to the antimicrobial peptides LL-37 and hBD2 as well as the antibiotic cephalexin. Taken together, these findings suggest that *L. crispatus* could have a beneficial effect on the host through co-aggregation with meningococci.

## Materials and methods

### Bacterial strains and growth conditions

All strains are listed in Supplementary Table S1. *N. meningitidis* strains FAM20 and M789 (serogroup C), MC58 (serogroup B), JB515 and ROU (both serogroup W135), Z6466 and Z2491 (serogroup A), and *N. meningitidis* FAM20 mutants ∆*pilT*,* ∆pilE*,* ∆siaD*,* ∆pilC1*,* ∆pilC2*,* ∆nafA*, ∆*lpxA*,* ∆pptB* and *∆pglH* have previously been described [8, 12, 18, 31–40]. *Lactobacillus crispatus* MV24, *Limosilactobacillus reuteri* ATCC PTA5289, *Lactobacillus gasseri* MV1, *Ligilactobacillus salivarius* LM9477, and *Lacticaseibacillus rhamnosus* Kx151A1 have also previously been described [41, 42]. *L. crispatus* ATCC33820 and ATCC33197 were purchased for this study (ATCC, Manassas, VA, USA). The term *Lactobacillus* is used in this paper for simplicity, but acknowledge the nomenclature changes in the genus as of 2020 [43]. Meningococcal strains were grown overnight on GC agar (Neugen Cultured Media, Lansing MI, USA) supplemented with 1% Kellogg’s solution [44]. Lactobacilli were cultured on solid Rogosa agar plates (Oxoid Inc, Hampshire, UK) for two days and in MRS medium (MRS; Oxoid Inc) overnight before the experiment. Bacteria were incubated in 5% CO_2_ at 37 °C. The medium used for the experiment was Dulbecco’s modified Eagle’s medium (DMEM, Thermo Fisher Scientific, Waltham, MA, USA) supplemented with 2 mM glutaMAX (Thermo Fisher Scientific) and 1 mM pyruvate (Thermo Fisher Scientific), and 1% fetal bovine serum (FBS; Sigma-Aldrich, Burlington, MA, USA).

### Coaggregation assay

Bacteria were grown as described above and transferred to prewarmed DMEM supplemented with 1% FBS. *N. meningitidis* were filtered through a 5-µm pore filter to break apart preexisting aggregates and stabilized for 1 h to allow microcolony formation. *N. meningitidis* was incubated with lactobacilli in a ratio of 1:1 at 10^7^ CFU/ml. The co-aggregation was visualized via live-cell imaging (Axiovert Z1, Zeiss, Oberkochen, Germany). Bacteria were added to a 24-well glass-bottom plate (MatTek, Ashland, MA, USA), incubated at 37 °C, 5% CO_2_, and observed over time, with images taken at 1 h and 2 h. A sedimentation assay was performed and analyzed as previously described by Kos et al. [45] in DMEM without FBS, and with the same bacterial concentration as in the live-cell imaging setting, and the optical density of the supernatant was checked at 2 h or at multiple time points.

### Adherence assay

The human epithelial cell line FaDu (ATCC HTB-43) was maintained in Dulbecco’s modified Eagle’s medium containing GlutaMax and pyruvate (DMEM; Thermo Fisher Scientific) and supplemented with 10% heat-inactivated fetal bovine serum (FBS; Sigma-Aldrich). Log phase *N. meningitidis* and lactobacilli were resuspended in DMEM supplemented with 1% FBS. FaDu cells were incubated with bacteria at a MOI of 100, and in ratio 1:1. After incubation for 2 h, unbound bacteria were removed by washing, and cells were lysed with 1% saponin in DMEM. Adherence was estimated by viable count with GC agar for *N. meningitidis* and solid Rogosa agar plates (Oxiod Inc) for lactobacilli.

### Pili Preparation

The pili preparation protocol was performed as previously described with some modifications [46]. Meningococci were grown overnight on one full GC plate, scraped off and suspended in 1 ml 0.15 M ethanolamine pH 10.5. Pili were sheared off by vortexing twice for 1 min and then centrifuged at 15 000 x g for 20 min at 22 °C. The supernatant (850 µl) was recovered, and 90 µl of ammonium sulfate-saturated 0.15 M ethanolamine was added slowly. After 30 min of incubation on ice, the pilus filaments were recovered by centrifugation at 21 000 x g for 20 min at 4 °C. The pellets were dissolved in 50 µl of PBS (pH 7.5). The protein concentration was tested by absorbance at 260/280 nm in a NanoDrop 8-Sample Spectrophotometer ND-8000 machine (Saveen Werner, Malmö, Sweden). Purity and size were analyzed by SDS-PAGE with β-mercaptoethanol (BME; Sigma-Aldrich), and the samples were heated at 95 °C and stained with Coomassie blue. For the uncropped gel, see the supplemental materials (Supplementary Fig. S1A). A Western blot using a pilus-antibody was performed to confirm the that the *~* 16 kDa band corresponds pilin (Supplementary Fig. S1B).

### Imaging of *Lactobacillus*-pili binding

*L. crispatus* MV24 and *L. gasseri* MV1 were cultured as described above. A total of 10^7^ CFU/ml bacteria in DMEM with 1% FBS were incubated for 1 h in 5% CO_2_ at 37 °C, centrifuged for 1 min, and resuspended in medium with 0.15 µg/µl or 0.025 µg/µl purified pili. Lactobacilli were incubated with pili for 30 min, washed in PBS and fixed with 4% paraformaldehyde (PFA; Sigma-Aldrich). To detect pili, the samples were treated with polyclonal rabbit anti-pili antibodies diluted 1:5000, washed three times in PBST (PBS with 1% Tween) and 0.3% BSA, and incubated with the secondary antibody Alexa 488-conjugated donkey anti-rabbit IgG (Invitrogen, Waltham, MA, USA) diluted 1:5000. Both antibodies were incubated at room temperature for 1 h.

To test effects of different enzymatic treatments, *L. crispatus* was first fixed in 4% PFA before incubation with 1 µg/µl of amylase, 1 µg/µl lipase, or 1 µg/µl proteinase K (all chemicals from Sigma-Aldrich) for 2 h at 37 °C. The enzymes were inactivated by 3 min of boiling, and the samples were washed three times in PBST before the addition of 0.15 µg/µl pili. The antibody-stained samples were washed 3x, resuspended in PBS, added to a 24-well glass-bottom plate (MatTek), and imaged under a fluorescence microscope (Axiovert Z1, Zeiss) or wide-field microscope (Widefield Axio Observer 7, Zeiss).

### Motility assay

The motility assay, developed by Turnbull and Whitchurch [47], was used with some modifications. *N. meningitidis* was filtered through a 5 μm pore filter and stabilized for 1 h to allow microcolony formation before the addition of *L. crispatus* or the control *L. gasseri*. *N. meningitidis* was used as a positive control, and non-motile lactobacilli were used as a negative control. After forming co-aggregative clusters for 2 h, 1 µl of the cell suspension was pipetted and injected into a 1% GC agar plate at three injection sites. The plates were incubated for 2 days before the plates were photographed with a ChemiDoc MP Imaging System (Bio-Rad, Hercules, CA, USA). The motility zones were quantified via Fiji software (ImageJ, version 2.1.0).

### Survival assays with LL-37, hBD2 and cephalexin

Bacteria were suspended in DMEM with 1% FBS at a ratio of 1:1 at 10^7^ CFU/ml. *N. meningitidis* were filtered through a 5-µm pore filter and stabilized for 1 h before the addition of lactobacilli together with 5 µM LL-37 (Innovagen AB, Lund Sweden), 5 µM hBD2 (Innovagen AB) or 0.5 µg/µl cephalexin (Sigma-Aldrich). The experiments were performed in a 96-well microtiter plate. After 3 h of incubation at 37 °C in 5% CO_2_, the bacteria were pipetted to dislodge the aggregates, serially diluted, and plated for viable counts on GC agar plates or Rogosa agar plates. To determine whether microcolonies and co-incubated aggregates were present during treatment, an experiment was performed with a 96-well plate containing glass lenses (MatTek) and visualized with an Axiovert Z1 microscope (Zeiss).

### Lactate detection

The detection of lactate in supernatants from *L. crispatus* and *L. gasseri* was performed using two lactate detection kits, MAK336 for D-lactate and MAK329 for L-lactate **(**Sigma-Aldrich). The assay was performed in accordance with the manufacturer’s recommendations. Lactobacilli were incubated for 4 h at 37 °C, 5% CO_2_ in DMEM with 1% FBS before being sterile-filtered through a 0.2-µm pore filter. The supernatant was collected and read at 565 nm using SpectraMax i3x (Molecular Devices, Silicon Valley, CA, USA).

### qPCR analysis

The bacteria were subsequently resuspended in DMEM supplemented with 1% FBS. Lactobacilli and *N. meningitidis* were and mixed at a ratio of 1:1 to 10^7^ CFU/ml each for 2 h. The samples were lysed and RNA was extracted with an RNeasy plus mini kit (Qiagen) according to manufacturer’s instructions. The RNA concentration and purity were measured using NanoDrop 8000. The RNA was converted into cDNA using superscript VILO mastermix (Thermo Fisher). qPCR analysis was conducted on a LightCycler480 system (Roche) using SYBR Green Master Mix (Roche). The qPCR program was as follows: initial denaturation at 95 °C for 10 min followed by 40 cycles of 95 °C for 10 s (denaturation), 50 °C for 20 s (annealing), 72 °C for 20 s (extension). Melt curve analysis: 95 °C for 5 s, 65 °C for 60 s, and 0.08 C/s until 95 °C was reached. The resulting values were normalized to those of the housekeeping gene *rpsJ*. The data are presented as the fold change relative to *N. meningitidis* without lactobacilli. The primers utilized are listed in SupplementaryTable S2.

### Statistics

An analysis of variance (ANOVA) was used to analyze differences between multiple groups. A Bonferroni post hoc correction was subsequently performed, and GraphPad Prism, version 9.4.1, was used for the analysis. P-values less than 0.05 were considered statistically significant.

## Results

### *Lactobacillus* crispatus co-aggregates with *N. meningitidis*

**Fig. 1:**
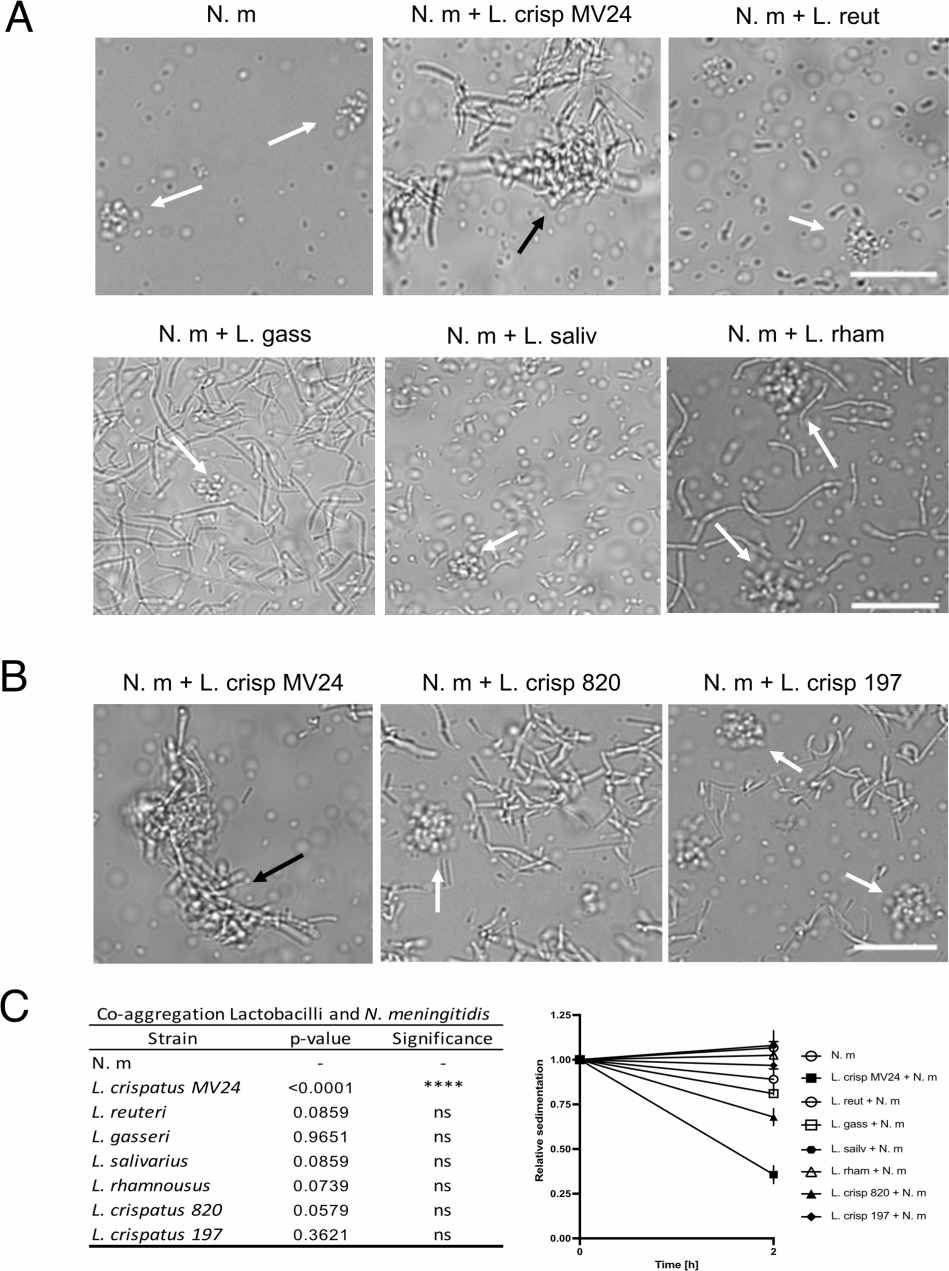
Co-aggregation between *N. meningitidis* FAM20 and *Lactobacillus* strains. **A** Co-incubation of *N. meningitidis* FAM20 (N. m) and different *Lactobacillus* species: L. crisp (*L. crispatus* MV24, L. reut (*L. reuteri* ATCC PTA5289), L. gass (*L gasseri* MV1), and L. saliv (*L. salivarius* LM9477), L. rham (*L. rhamnosus* Kx151A1). **B** Co-incubation of *N. meningitidis* FAM20 (N. m) with three *L. crispatus* strains: L.crisp MV24, L. crisp 820 (ATCC33820) or L. crisp 197 (ATCC33197). The white arrows indicate meningococcal microcolonies; the black arrows indicate co-aggregative clusters. The aggregation was imaged at 40x magnification with an Axiovert Z1 microscope (Zeiss). Scale bar 20 μm. **C** Sedimentation assay. Optical density was measured in the supernatant at the beginning and after 2 h of incubation. Values are normalized to the time point at 0 h, and compared with N. m alone. The error bars represent the standard deviation. Three technical repeats were performed. ****p < 0.001; ns, non-significant

*Lactobacillus* species are part of the human microbiota and come in direct contact with pathogenic bacteria. Here, we examined the interaction between *N. meningitidis* FAM20 and different *Lactobacillus* strains using live-cell microscopy. Bacteria were co-incubated at a ratio of 1:1 for 2 h. Microscopy images revealed that *L. crispatus* MV24 bound to meningococcal microcolonies in a co-aggregative manner (Fig. 1A, black arrow). *L. crispatus* bound to meningococci during the initial microcolony formation, resulting in aggregates that were not rounded or spherical, but instead exhibit a more irregular shape (Supplementary Fig. S2). The other four tested *Lactobacillus* species; *Limosilactobacillus reuteri*, *Lactobacillus gasseri*, *Ligilactobacillus salivarius* and *Lacticaseibacillus rhamnosus*, did not co-aggregate with *N. meningitidis* (Fig. 1A, white arrows), and therefore left the meningococcal microcolonies undisturbed. None of the *Lactobacillus* strains inhibited *N. meningitidis* attachment to pharyngeal FaDu cells, instead *L. crispatus* increased attachment since aggregates fall faster towards the cell surface (Supplementary Fig. S3), To investigate whether other *L. crispatus* strains also co-aggregate with *N. meningitidis*, we included two additional strains, *L. crispatus* 33,820 and *L. crispatus* 33,197, which both failed to co-aggregate with meningococcal microcolonies, indicating strain specificity (Fig. 1B). To further confirm the co-aggregation of *L. crispatus* MV24 with *N. meningitidis*, we performed a sedimentation assay in which bacteria were co-incubated together or alone for 2 h, and the optical density of the uppermost part of the liquid was measured to estimate the sedimentation. Co-incubated bacteria were compared with bacteria alone. The sedimentation assay revealed that *L. crispatus* MV24 significantly co-aggregated with *N. meningitidis*, whereas all the other strains failed to co-aggregate (Fig. 1C). Taken together, L. *crispatus* MV24, but not the other *Lactobacillus* strains tested, co-aggregated with *N. meningitidis* microcolonies.

### Meningococcal microcolony formation is linked to co-aggregation with *L. crispatus*

**Fig. 2:**
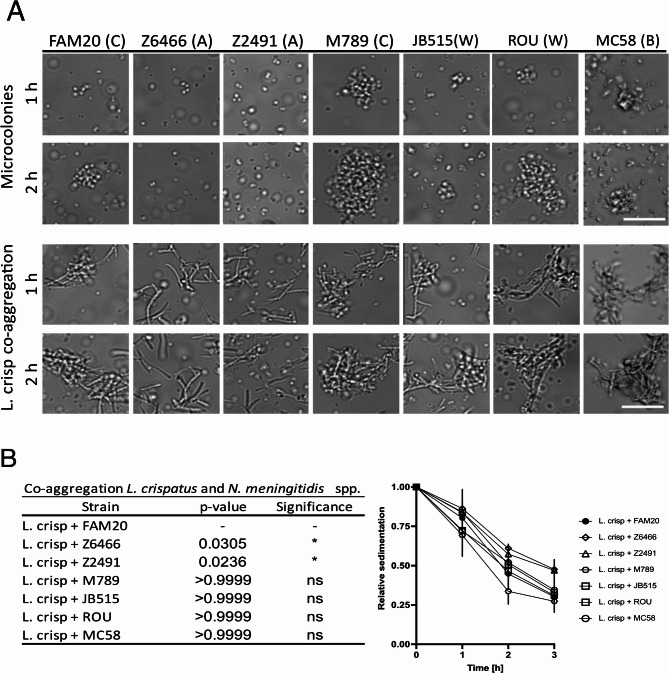
Co-aggregation of *L. crispatus* MV24 with different meningococcal strains. (**A**) Upper two rows: Microcolony formation of *N. meningitidis* at 1 h and 2 h post incubation. Lower two rows: co-incubation of *L. crispatus* MV24 with *N. meningitidis* strains for 1 h and 2 h. *N. meningitidis* strains with serogroup in parentheses: FAM20 (C), Z6466 (A), Z2491 (A), M789 (C), JB515 (W-135), ROU (W-135) and MC58 (B). Bacteria were imaged with an Axiovert Z1 microscope (Zeiss) at 40x magnification. Scale bar 20 μm. (**B**) Sedimentation assay. Optical density was measured at the start of incubation (0 h) and after 1 h, 2 h and 3 h of co-incubation. Values are normalized with respect to the time point at zero hours, and the error bars represent the standard deviation. Three technical repeats were performed and statistics at 3 h. *p* < 0.05, ns non-significant

Since only one of the tested *Lactobacillus* strains bound to meningococci, we next assessed whether the co-aggregation was also specific for just one meningococcal strain and capsular serogroup. Six additional meningococcal strains were tested, two with capsules belonging to serogroup A (Z6466 and Z2491), one from serogroup C (M789), two from serogroup W-135 (JB515 and ROU), and one from serogroup B (MC58). The bacteria were observed under a microscope as described previously, and after one hour, meningococci alone started to form microcolonies. As shown in the upper two panels of Fig. 2A, there was a difference in the ability and rate of the strains to form microcolonies, with M789, ROU and MC58 forming larger aggregates faster and Z6466 and Z2491 not forming microcolonies. Strain JB515 had an intermediate ability to form microcolonies. When the meningococcal strains were incubated together with *L. crispatus* MV24, only the meningococci able to form microcolonies co-aggregated with *L. crispatus* (Fig. 2A, two lower panels). Aggregation was confirmed in a sedimentation assay, where the strains that formed microcolonies and co-aggregated with *L. crispatus* sedimented faster than strains that did not form microcolonies (Fig. 2B). These findings indicate that microcolony formation is necessary for co-aggregation with *L. crispatus.*

### *L. crispatus* fails to aggregate with a meningococcal Δ*pilE* mutant but hyper-aggregates with a Δ*pilT* mutant

**Fig. 3:**
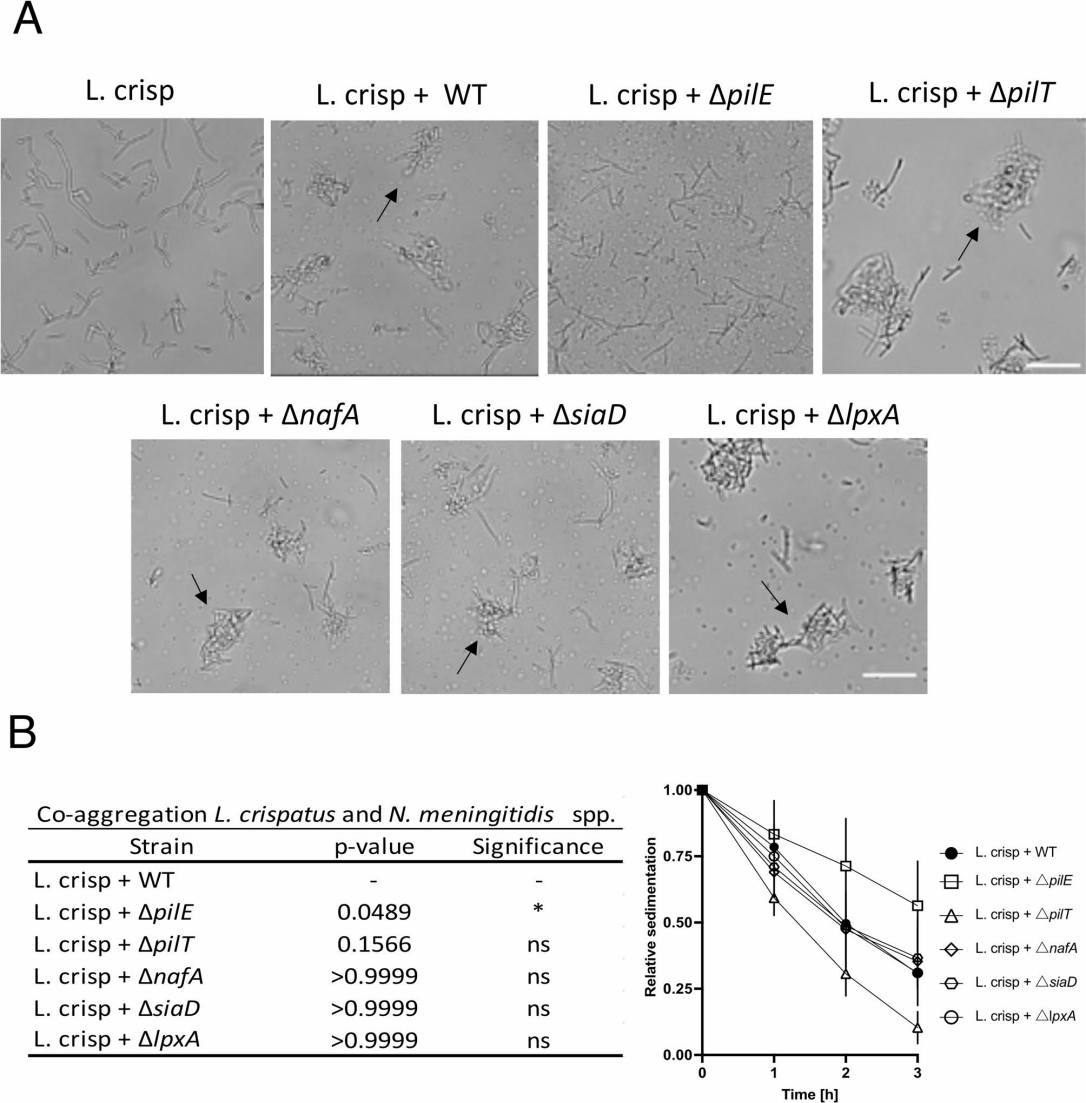
*L. crispatus* MV24 fails to co-aggregate with meningococcal mutants lacking PilE. **A** Live-cell microscopy images of L. crisp (*L. crispatus* MV24), WT (*N. meningitidis* FAM20), and *N. meningitidis* deletion mutants: Δ*pilE*, ∆*pilT*, Δ*nafA*, Δ*siaD*, and Δ*lpxA*. The black arrows indicate co-aggregative clusters. The aggregation was imaged at 40x magnification with an Axiovert Z1 microscope (Zeiss). Scale bar 20 μm. **B** Sedimentation assay. Optical density measurements were taken at 0, 1, 2, and 3 h after the addition of lactobacilli. Values are normalized with respect to the time point at zero hours, and the error bars show the standard deviation. Four technical repeats were performed and statistics at 3 h. *p* < 0.05, ns non-significant

To identify the meningococcal factors that influence the co-aggregation with *L. crispatus*, we tested a set of isogenic mutants of *N. meningitidis*. Wild-type *N. meningitidis* and five different mutants, Δ*pilE*,* ∆pilT*,* ∆nafA*, *∆siaD*, and *∆lpxA*, were co-incubated with *L. crispatus* for 2 h. The Δ*nafA* and ΔpilT mutants are both hyper-aggregative and remain in microcolonies due to the lack of the anti-aggregative factor NafA and the pilus retractive ATPase PilT, respectively. The capsule-deficient mutant *ΔsiaD*, the LPS mutant Δ*lpxA*, and the pilus-deficient Δ*pilE* mutant lack important surface factors. Four of the mutants, *∆pilT*, Δ*nafA*,* ∆siaD*,* and ∆lpxA*, co-aggregated with *L. crispatus* MV24, in a manner similar to that of the wild-type strain, whereas the meningococcal *ΔpilE* mutant did not co-aggregate with lactobacilli (Fig. 3A). In a sedimentation assay, co-incubation of *N. meningitidis* Δ*pilE* with *L. crispatus* resulted in the lowest degree of sediment, whereas *L. crispatus* and Δ*pilT* resulted in the fastest sedimentation (Fig. 3B). These findings suggest that pili expression is required for co-aggregation between *N. meningitidis* and *L. crispatus.*

### *L. crispatus* binds to meningococcal pili

**Fig. 4:**
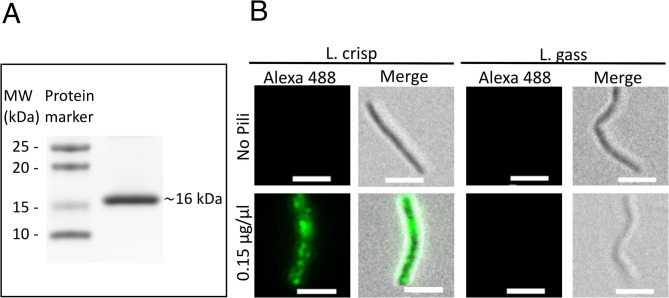
Purified meningococcal pili bind to *L. crispatus* but not to *L. gasseri.***A** Purified *N. meningitidis* FAM20 pili were separated via SDS-PAGE and stained with Coomassie blue. Size marker at the left. **B** Binding of meningococcal pili to *L. crispatus* imaged via fluorescence microscopy. *L. crispatus* MV24 (L. crisp) or *L. gasseri* MV1 (L. gass) at 10^7^ CFU/ml were incubated in DMEM supplemented with 1% FBS for 1 h before incubation with 0.15 µg/µl of pili preparation or a control mixture with no added pili for 30 min. The samples were then washed, fixed in 4% PFA and subsequently stained with primary anti-pili antibody and secondary Alexa 488-conjugated anti-IgG. L. crisp (*L. crispatus* MV24) and L. gass (*L. gasseri* MV1). Images were taken at 100x magnification with an Axiovert Z1 microscope (Zeiss) with Alexa 488, and merged with brightfield images. Scale bar, 5 μm. The merged images represent a merge of the fluorescence and brightfield images

Pili are required for successful microcolony formation and it is possible that *L. crispatus* may interact with meningococcal pili during co-aggregation. To test whether the meningococcal pili could bind to lactobacilli, we purified pili of the *N. meningitidis* strain FAM20, which is a Class II pili with a 16 kDa PilE subunit (Fig. 4A). Pili were mixed with *L. crispatus* or the non-aggregative *L. gasseri* for 30 min and then stained with pili antibodies and an Alexa Fluor 488 conjugated secondary antibody, and the binding was analyzed by fluorescence microscopy. Pili were detected bound to *L. crispatus*, but not to the negative control *L. gasseri* (Fig. 4B), suggesting that pili are involved in the interaction with *L. crispatus*. Furthermore, the control with no pili added produced no signal, verifying that the pili bound specifically to *L. crispatus*.

### *L. crispatus* binds to both class I and class II pili possibly via a lipoprotein

**Fig. 5:**
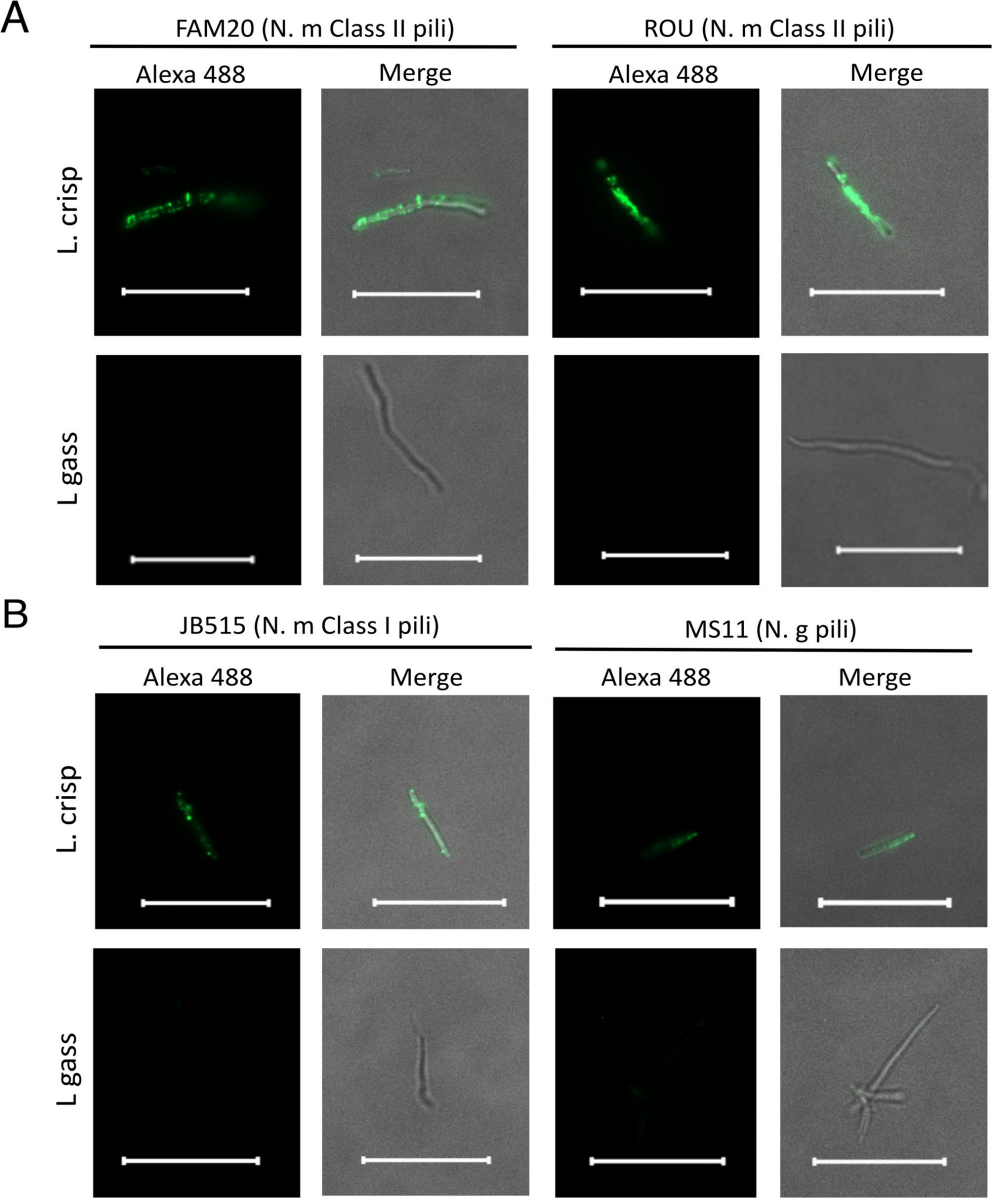
*L. crispatus* MV24 binds to both Class I and Class II pili. Binding of meningococcal Class I and Class II pili to lactobacilli as imaged by fluorescence microscopy. L. crisp (*L. crispatus* MV24) and L. gass (*L. gasseri* MV1) were incubated with 0.025 µg/µl of pili preparation or medium control for 30 min, fixed in 4% PFA and stained with primary anti-pili antibody and secondary Alexa 488-conjugated anti-IgG. **A** Pili preparation from *N. meningitidis* strains FAM20 and ROU (Class II pili). **B** Pili preparation from *N. meningitidis* strains JB515 (Class I) and *Neisseria gonorrhoeae* (MS11). Images were collected at 40x magnification with a fluorescence microscope (Widefield Axio Observer 7, Zeiss), and merged with brightfield images. Scale bar, 10 μm. The merged images represent a merge of the fluorescence and brightfield images

**Fig. 6:**
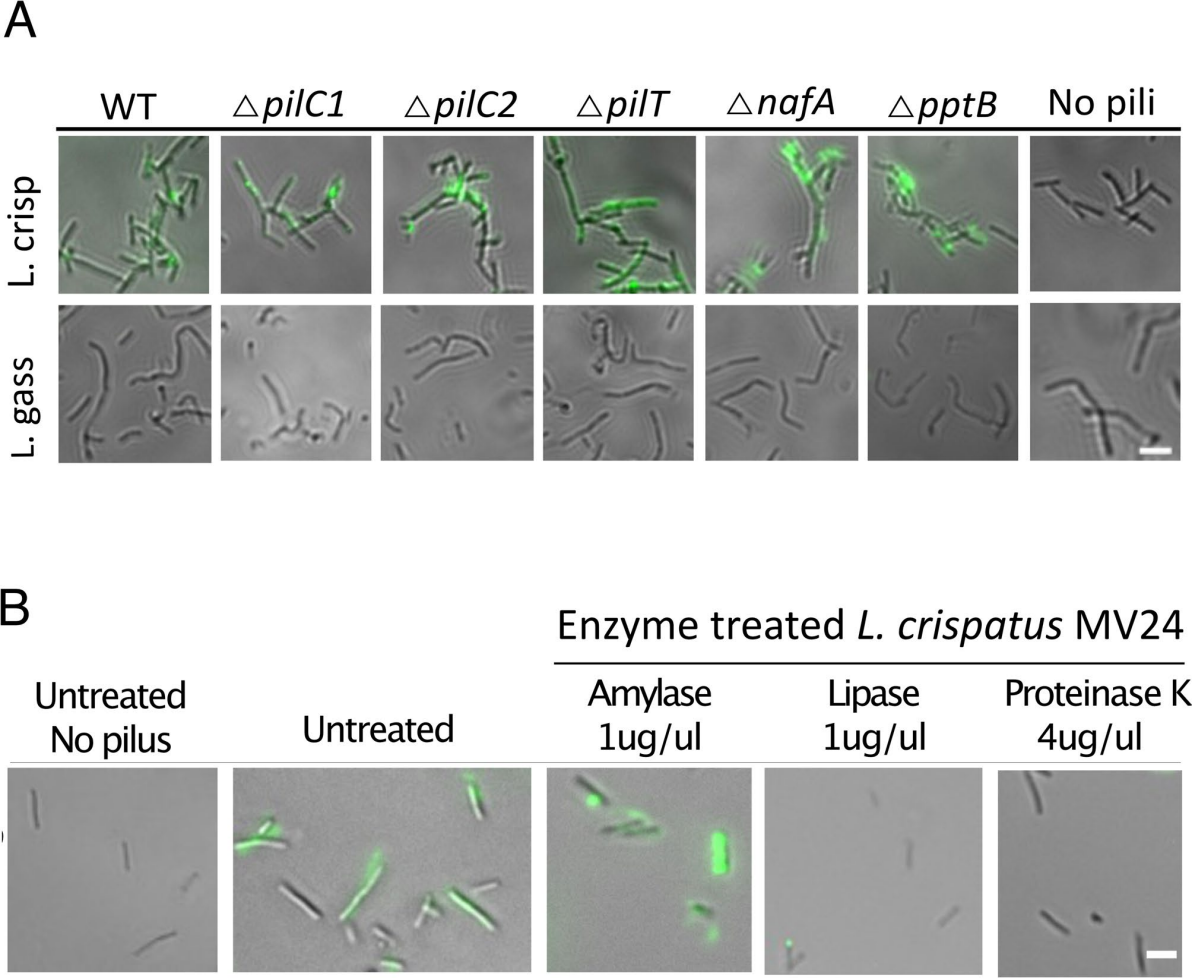
*L. crispatus* loses pili-binding ability after treatment with lipase and proteinase K. **A** Binding of pili isolated from wild-type (WT) *N. meningitidis* and its isogenic *pilC1*, *pilC2*, *pilT*, *nafA*, and *pptB* mutants to lactobacilli. *L. crispatus* MV24 (L. crisp) and *L. gasseri* MV1 (L. gass) were incubated in DMEM supplemented with 1% FBS for 1 h, followed by 30 min of 0.025 µg/µl of pili preparation before fixation in 4% PFA. The samples were stained with primary antibody (rabbit anti-pili serum against PilE) and secondary antibody (Alexa 488-conjugated anti-rabbit IgG) and analyzed withan Axiovert Z1(Zeiss) fluorescence microscope at 100x magnification. Scale bar, 5 μm. **B** *L. crispatus* was fixed with 4% PFA, treated with 1 µg/µl of amylase, lipase, or proteinase K, washed three times and incubated with 0.15 µg/µl pili. The experiment was performed with primary polyclonal antibodies against pili and the secondary antibody Alexa 488-conjugated anti-rabbit IgG. The samples were analyzed via a fluorescence microscope Axiovert Z1 (Zeiss). Magnification 100x. Scale bar, 5 μm

*N. meningitidis* strains can express one of two types of pili, Class I (approximately 20 kDa and similar to gonococcal PilE) or Class II (smaller PilE of approximately 16 kDa). To analyze whether *L. crispatus* could bind both of these pilus types, we purified pili from *N. meningitidis* FAM20 (Class II), ROU (Class II), JB515 (Class I) and *N. gonorrhoeae* MS11 and analyzed their binding by fluorescence microscopy. Both Class II (Fig. 5A), and Class I, as well as the gonococcal pili (Fig. 5B), bound strongly to *L. crispatus*. To further examine whether we could find pilus-associated components involved in the binding to *L. crispatus*, we purified pili from a ∆*pilT* mutant (pilus retraction deficient), ∆n*afA* mutant (deficient in aggregation), ∆*pptB* mutant (lacking pilin phosphotransferase B PptB), and ∆*pilC1* and ∆*pilC2* mutants (pilus tip adhesins). All these purified pili bound to *L. crispatus*, but not to *L. gasseri* (Fig. 6A). The ability of pili to bind to target molecules can be altered by post-translational modification. However, co-incubation of meningococci with *L. crispatus* or *L. gasseri* did not affect the expression of genes involved in pilus glycosylation (*pgl*-genes) or the phosphotransferase B gene (*pptB*) adding phosphoglycerol to PilE (Supplementary Fig. S4A). In addition, a ∆*pglH*-mutant lacking pilin glycosylation, co-aggregated with *L. crispatus* similar to wild-type *N. meningitidis* (Supplementary Fig. S4B). To shed light on the nature of the *Lactobacillus* component that binds to pili, we treated *L. crispatus* with amylase, lipase, or proteinase K. Interestingly, pili did not bind to *L. crispatus* treated with lipase or proteinase K, while pili still bound to lactobacilli treated with amylase (Fig. 6B). In summary, *L. crispatus*, but not *L. gasseri*, binds to both Class I and Class II meningococcal pili and to all tested pili preparations from mutants lacking pilus biogenesis functions, but this pili-binding ability is lost after treatment with lipase and proteinase K, indicating that pili might bind to a lipoprotein.

### *L. crispatus* decreases the *N. meningitidis* motility zone in soft agar plates

**Fig. 7:**
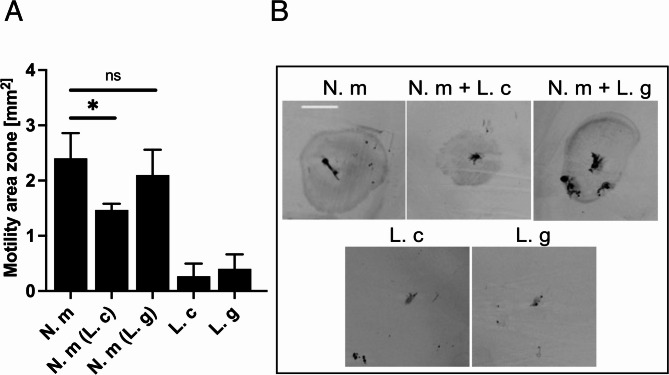
*L. crispatus* reduces the *N. meningitidis* motility zone ion agar plates. *N. meningitidis* FAM20 (N. m) was incubated alone or co-incubated with *L. crispatus* MV24 (L. c) and *L. gasseri* MV1 (L. g) for 2 h before 1 µl was injected into a 1% GC agar plate. Single cultures of lactobacilli were used to verify that the lactobacilli were nonmotile. After 2 days of incubation, the plates were imaged with a ChemiDoc MP Imaging System (Bio-Rad). **A** Quantitative data on the motility area zone. **B** Representative images of the motility zone area. Scale bar, 0.5 mm. Three technical repeats were performed with five plates per bacterial combination. The bars represent the standard deviation. *p < 0.05; ns, nonsignificant; negative control bars were not tested for significance

*N. meningitidis* can move via the type IV pilus-mediated retraction and extension. To assess whether co-aggregation with lactobacilli affects their ability to move, a soft-agar motility assay was performed. *N. meningitidis* were incubated alone or together with *L. crispatus* or *L. gasseri* for 2 h and transferred to a motility agar plate. Meningococci incubated with *L. crispatus* presented a reduced motility area, whereas those incubated with *L. gasseri* presented no effect compared with *N. meningitidis* alone (Fig. 7A and B). Single cultures of lactobacilli were used to verify that the lactobacilli were nonmotile. Because lactobacilli release high levels of lactate, which might affect meningococcal movement through chemotaxis, we measured the production of D-lactate and L-lactate by *L. crispatus* and *L. gasseri*. However, no significant differences were observed between the two strains; both strains produced L- and D-lactate at similar levels (Supplementary Fig. S5). Thus, co-aggregation of *N. meningitidis* and *L. crispatus* reduced the motility of meningococci.

### *L. crispatus* co-aggregation with *N. meningitidis* affects survival in antimicrobial agents

**Fig. 8:**
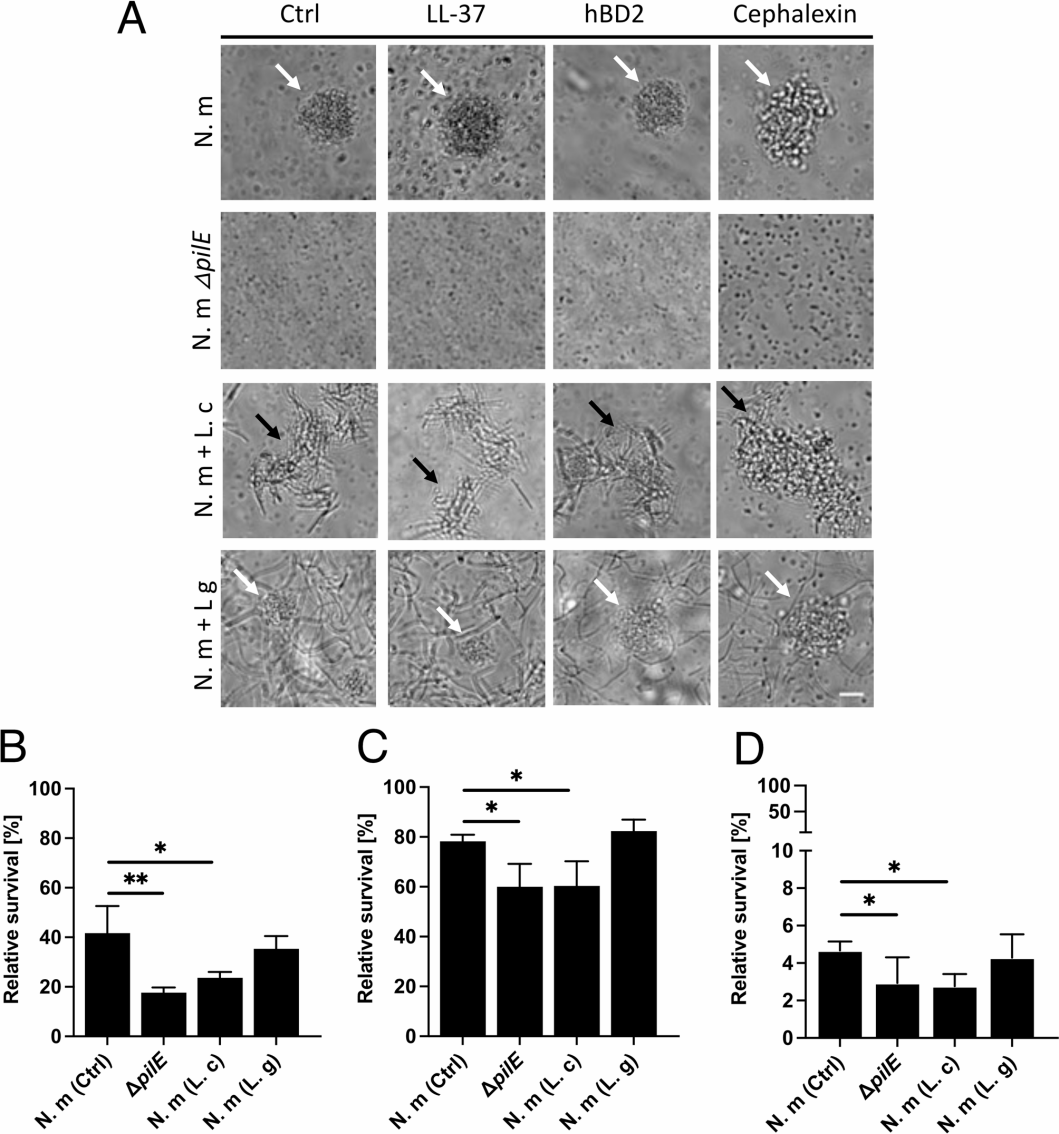
*L. crispatus* co-aggregation with *N. meningitidis* affects survival in antimicrobial agents. **A** Microscopy images showing microcolony formation and co-aggregation between meningococci and lactobacilli in the presence of LL-37 (5 µM), hBD2 (5 µM) or cephalexin (0.5 µg/µl. After 3 h, bacteria were imaged under an Axiovert Z1 Zeiss microscope at 40x magnification. Scale bar, 10 μm. The nonpiliated mutant (N. m Δ*pilE*) was used as a negative control. Bacterial strains: N. m (*N. meningitidis* FAM20), L. c (*L. crispatus* MV24), and L. g (*L. gasseri* MV1). **B–D** Survival assays of *N. meningitidis* co-incubated with lactobacilli in the presence of (**B**) 5 µM LL-37, (**C**) 5 µM hBD2 or (**D**) 0.5 µg/µl cephalexin for 3 h. Bacteria were plated for viable counts. Three technical repeats were performed. Percentage relative survival was calculated as treated/untreated. The bars represent the standard deviation. **p < 0.01; *p < 0.05; unmarked bars are considered nonsignificant

Next, we aimed to assess whether the co-aggregation between meningococci and *L. crispatus* had an effect on the ability of the bacteria to survive antimicrobial peptides and antibiotics. First, we examined the ability to still form microcolonies and co-aggregate in the presence of LL-37, hBD2, and cephalexin. As shown in Fig. 8A, in the upper two panels, *N. meningitidis* wild type formed microcolonies after incubation for 2 h with LL-37, hBD2 and cephalexin, whereas the *pilE* mutant did not. The *pilE* mutant was also more sensitive to LL-37 (Fig. 8B), hBD2 (Fig. 8C) and cephalexin (Fig. 8D), demonstrating the importance of microcolonies in surviving exposure to antimicrobial agents. In co-incubation assays, *N. meningitidis* and *L. crispatus* co-aggregated, whereas *L. gasseri* still failed to co-aggregate in the presence of the antimicrobials (Fig. 8A, two lower panels). *N. meningitidis* that co-aggregated with *L. crispatus* was more sensitive to treatment with LL-37 (Fig. 8B), hBD2 (Fig. 8C) or cephalexin (Fig. 8D) compared to the sensitivities of meningococci incubated with *L. gasseri* or the medium control. Lactobacilli alone were not killed by the antimicrobial agents (Supplementary Fig. S6). These data suggest that *L. crispatus* co-aggregation interferes with the ability of *N. meningitidis* to form functional microcolonies, leading to increased susceptibility to antimicrobials.

## Discussion

Bacterial pathogens that colonize epithelial linings must interact with and respond to signals and factors from the normal flora community. *Lactobacillus* species are important inhabitants of the epithelial surfaces of the human nasopharynx and pharynx. These non-harmful bacteria are known to have numerous positive effects that counteract the ability of pathogens to establish themselves. However, although many studies have shown beneficial effects, much remains to be understood about the role of lactobacilli. In this study, we investigated the interaction between lactobacilli and the nasopharyngeal commensal *N. meningitidis*, which frequently asymptomatically colonizes 5–10% of the human population but only occasionally causes severe disease. Among the *Lactobacillus* species tested, only one strain, *L. crispatus* MV24, could co-aggregate with meningococci. Furthermore, *N. meningitidis* bound to *L. crispatus* during microcolony formation, which prevented proper microcolony formation and normal dispersal. Additionally, movement within the microcolonies decreased when the meningococci co-aggregated with *L. crispatus*. Although its biological role is not fully understood, the ability to undergo microcolony dispersal is thought to be a necessary step for meningococcal invasion of host cells as well as for spreading to new sites and hosts [48–50].

*N. meningitidis* lacking the *pilE* gene, which encodes the major pilus subunit, failed to aggregate with *L. crispatus*, whereas a *pilT* mutant, which exhibits a hyper-aggregative phenotype, co-aggregated heavily with *L. crispatus*. This finding led us to isolate meningococcal pili and assess their interaction with *L. crispatus* MV24 and *L. gasseri* MV1. Fluorescence microscopy revealed that the purified pili bound to *L. crispatus* but failed to bind to the non-aggregative *L. gasseri*. Both Class I and Class II pili bound to *L. crispatus*, suggesting that the binding region may not be located within the hypervariable domain that distinguishes these pili types. Pili from meningococcal mutants lacking the pilus adhesin genes *pilC*, *pptB* (which adds phosphoglycerol to PilE), *nafA* (an anti-aggregative factor) and *pilT* all aggregated well with *L. crispatus*. These results suggest that a component not tested here or a conserved region of PilE found on both Class I and Class II pili may interact with *L. crispatus*. *L. crispatus* co-aggregated with meningococcal strains of different serogroups, except two serogroup A strains. These strains did not form microcolonies, suggesting that they either did not express pili or expressed non-aggregative pili, both of which support the necessity of microcolony formation for co-aggregation with *L. crispatus*. It is also possible that the serogroup A capsule interfere with the co-aggregation. The pili preparations contained some minor proteins, either co-purifying or attached as part of the pilus (Supplementary Fig S1). We can not exclude that these minor co-purifying components may contribute to binding to *L. crispatus*. However, to identify and determine their contribution is beyond the scope of this study. In the future, it would be interesting to identify the possible involvement of these minor components. The exact domain of the pilus that binds to *L. crispatus* remains to be identified.

Type IV pili are not exclusive to pathogenic *Neisseria*, homologous Tfp variants are also present in respiratory pathogens such as *Moraxella catarrhalis* and *Pseudomonas aeruginosa*, as well as in *Escherichia coli* associated with bacterial vaginosis [51]. These pathogens may encounter *L. crispatus* in the vaginal or nasopharyngeal mucosa. Although this lies beyond the scope of the present study, it would be interesting to evaluate *L. crispatus* pilus-mediated aggregation with other pathogens, to determine whether this mechanism is general and broadly applicable across multiple bacterial species.

It is becoming increasingly clear that interactions between commensal and pathogenic bacteria can influence the outcome of an infection. Recently, a commensal *Neisseria* species was shown to co-localize with *N. meningitidis*. In a paper by Custodio et al., it was demonstrated that *Neisseria cinerea* can form microcolonies, and impair microcolony formation and colonization of meningococci on epithelial cells [52]. Although *N. cinerea* expresses pili, these pili are not involved in the interaction with meningococci. Additionally, meningococcal motility was impaired by interaction with *N. cinerea.* Here, we found that meningococcal movement was impaired in the presence of *L. crispatus* but remained unaffected when meningococci were co-incubated with the non-aggregative *L. gasseri* strain. Motility plays a crucial role in pathogenesis and virulence, as non-motile bacteria are less likely to invade into host cells, and are impaired in the attachment process [53, 54]. We have previously shown that pre-incubation of host cells with *L. crispatus* enhances meningococcal uptake by epithelial cells via caveolin-mediated endocytosis, leading to increased destruction of the pathogen [55]. The co-aggregation shown in this study could represent an additional beneficial effect on the host, with *L. crispatus* trapping meningococci in large clusters that sediment faster onto the host, allowing their uptake and intracellular killing.

LL-37 is synthesized by, for example, epithelial cells and neutrophils, whereas hBD2 is expressed in airway epithelial cells and keratinocytes [56, 57], and both reduce the survival of pathogenic *Neisseria* strains [58]. In this work, co-aggregation with *L. crispatus* increased the meningococcal sensitivity to antimicrobial peptides. Both LL-37 and hBD2 had stronger killing effects on meningococci co-aggregated with *L. crispatus* than on those co-incubated with *L. gasseri*. The survival of meningococci during exposure to LL-37 or hBD2 during colonization is crucial to establish themselves and invade host tissue. Additionally, antibiotic treatment with cephalexin eliminated meningococci that co-aggregated with *L. crispatus* more efficiently than non-aggregated meningococci. A reason for this could be impaired microcolony fluidity. In pathogenic *Neisseria*, the attractive force of tfp-tfp changes the microcolony into a more solid or liquid phase, which affects susceptibility to antibiotics and antimicrobial peptides (AMPs) [20, 59]. Enzymatic treatment of *L. crispatus* with lipase or proteinase K removed the ability to bind to pili, indicating that the binding molecule may have lipoprotein-like characteristics. In future experiments, identifying the *Lactobacillus*-derived molecule responsible for binding to meningococcal pili and evaluating whether this molecule can prevent or inhibit pilus-associated functions in meningococci would be interesting. In summary, this study provides new insights into host-microbe interactions, demonstrating that an *L. crispatus* strain can co-aggregate with *N. meningitidis* and reduce its survival in the presence of AMPs and antibiotics as well as limit motility and the formation of proper microcolonies, all of which could help host clearance of the pathogen. These findings suggest that *L. crispatus* may influence *N. meningitidis* through mechanisms independent of the host immune response.

## Conclusions

In this study, we assessed the binding properties of lactobacilli to *N. meningitidis* and identified one specific strain of *L. crispatus* that strongly co-aggregates with the pathogen. The binding of *L. crispatus* was related to the ability of *N. meningitidis* to form microcolonies and express pili, and we confirmed that *L. crispatus* binds to Type IV pili, including both Class I and Class II variants. Co-aggregation disrupts key pathogenic behaviors of *N. meningitidis*, impairing its motility and ability to form organized microcolonies. As a result, the pathogen becomes more susceptible to host antimicrobial agents, such as antibiotics and antimicrobial peptides, suggesting a protective role of *L. crispatus* in host-pathogen interactions.

## Supplementary Information

Supplementary Material 1.

## Data Availability

The datasets used and/or analyzed during the current study are available from the corresponding author upon reasonable request.
