# Supplementary material for: Pilus-mediated co-aggregation with Lactobacillus crispatus increases meningococcal susceptibility to antimicrobial agents by interfering with microcolony formation
Source: BMC Microbiol. 2025 Jul 30;25:467. doi: 10.1186/s12866-025-04201-2 (PMC12312235; doi:10.1186/s12866-025-04201-2)
Supplement: Supplementary file 1 — Supplementary Material 1. [file 12866_2025_4201_MOESM1_ESM.pdf]

Supplementary Material

Pilus-mediated co-aggregation with *Lactobacillus crispatus* increases meningococcal susceptibility to antimicrobial agents by interfering with microcolony formation

Kenny Lidberg[1], Sarah Pilheden[1], Samuddi Nawarathne[1], Katharina Rauscher[1], Ann-Beth Jonsson[1#]

[1] *Department of Molecular Biosciences, The Wenner-Gren Institute, Stockholm University, SE-10691 Stockholm, Sweden*

[#] Corresponding author: Ann-Beth Jonsson, E-mail: ann-beth.jonsson@su.se

FIGURE S1

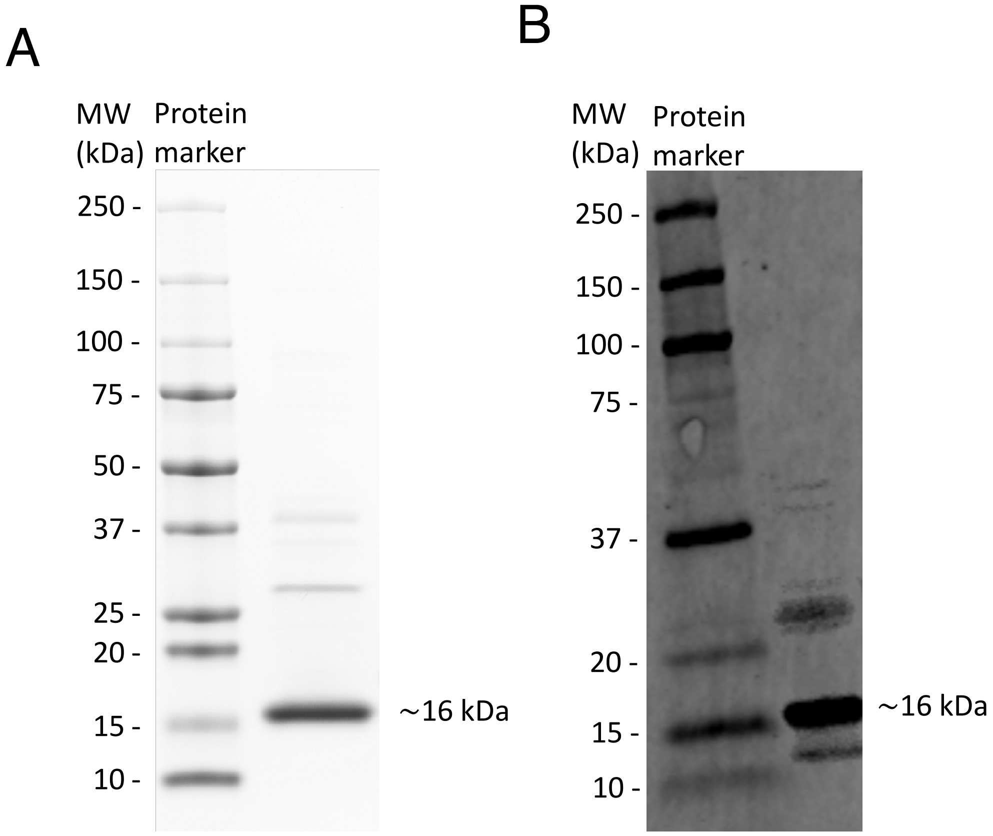

Fig. S1 Pili preparation and uncropped gel and western blot.

(A) SDS-PAGE of pili preparation from *N. meningitidis* FAM20 stained with Coomassie blue. (B) Western blot with pili preparation from *N. meningitidis* strain FAM20. The protein marker ladder is to the left.

FIGURE S2

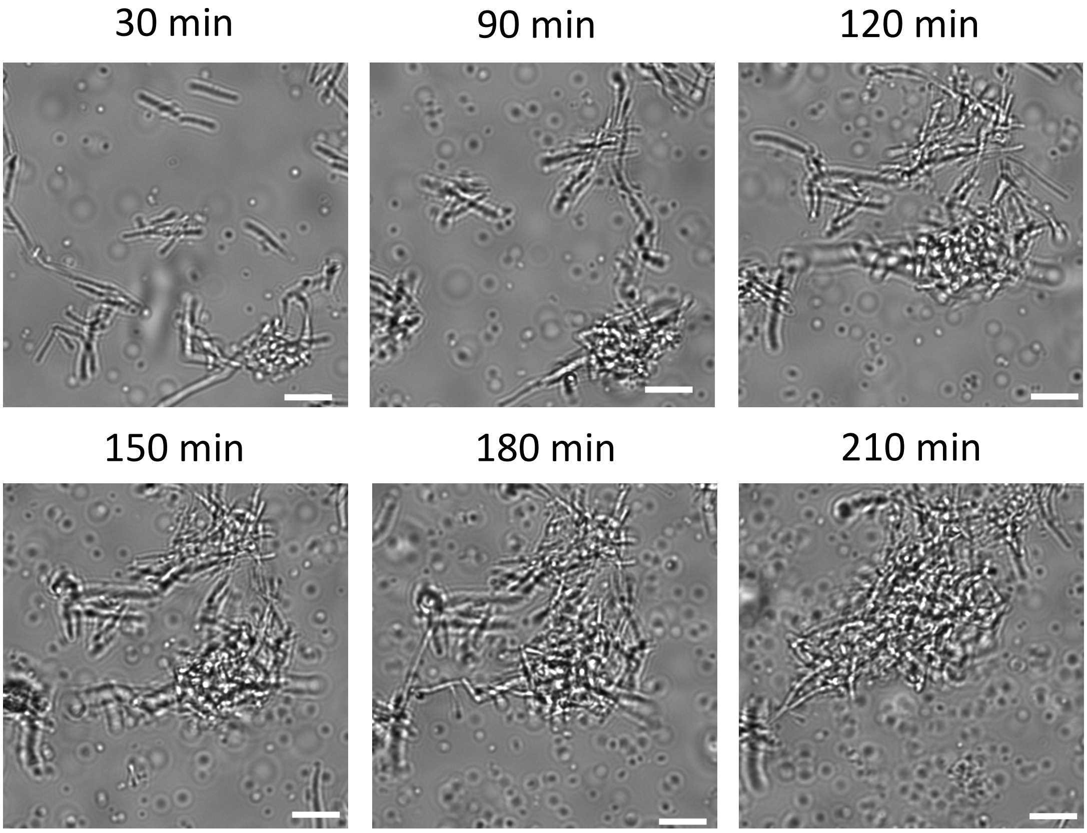

Fig. S2 *L. crispatus* and *N. meningitidis* aggregation over time.

Microscopy images showing co-aggregation between meningococci and *L. crispatus* MV24. Time point zero was set after the addition of *L. crispatus*. All images were taken at the same coordinates and follow the same cluster over time. Bacteria were imaged under an Axiovert Z1 Zeiss microscope at 40x magnification. Scale bar, 10 µm.

FIGURE S3

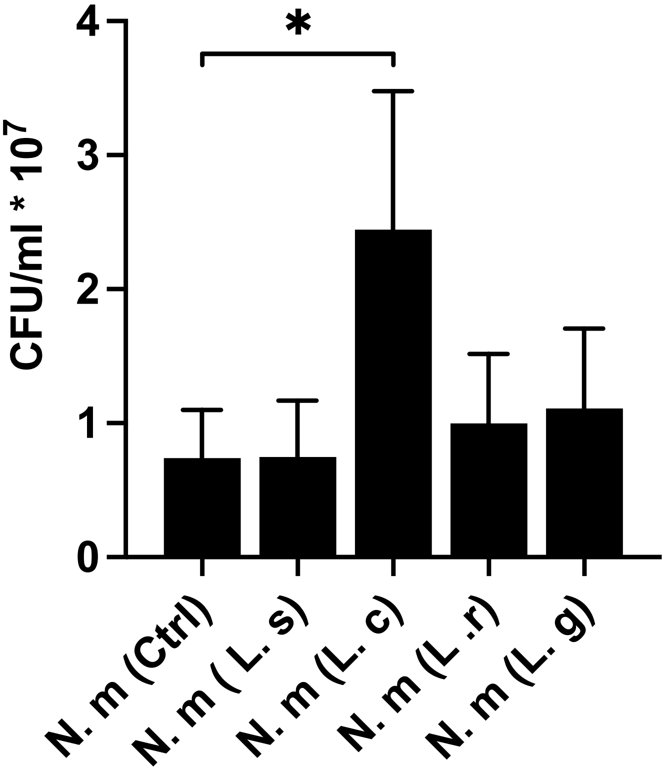

Fig. S3 Meningococci attachment to host cells in co-culture with lactobacilli.

Attachment to FaDu pharyngeal cells of *N. meningitidis* (N. m) in the presence or absence of lactobacilli. *L. salivarius* (L. s), *L. crispatus* (L. c), *L. reuteri* (L. r), *L. gasseri* (L. g), or only meningococci (Ctrl). All bacteria were added simultaneously and then incubated for 2 h. Bound meningococci were plated to determine the colony-forming units per ml (CFU/ml). Four technical repeats were performed. The bars represent the standard deviation. ****$p < 0.001$; unmarked bars are considered nonsignificant.

FIGURE S4

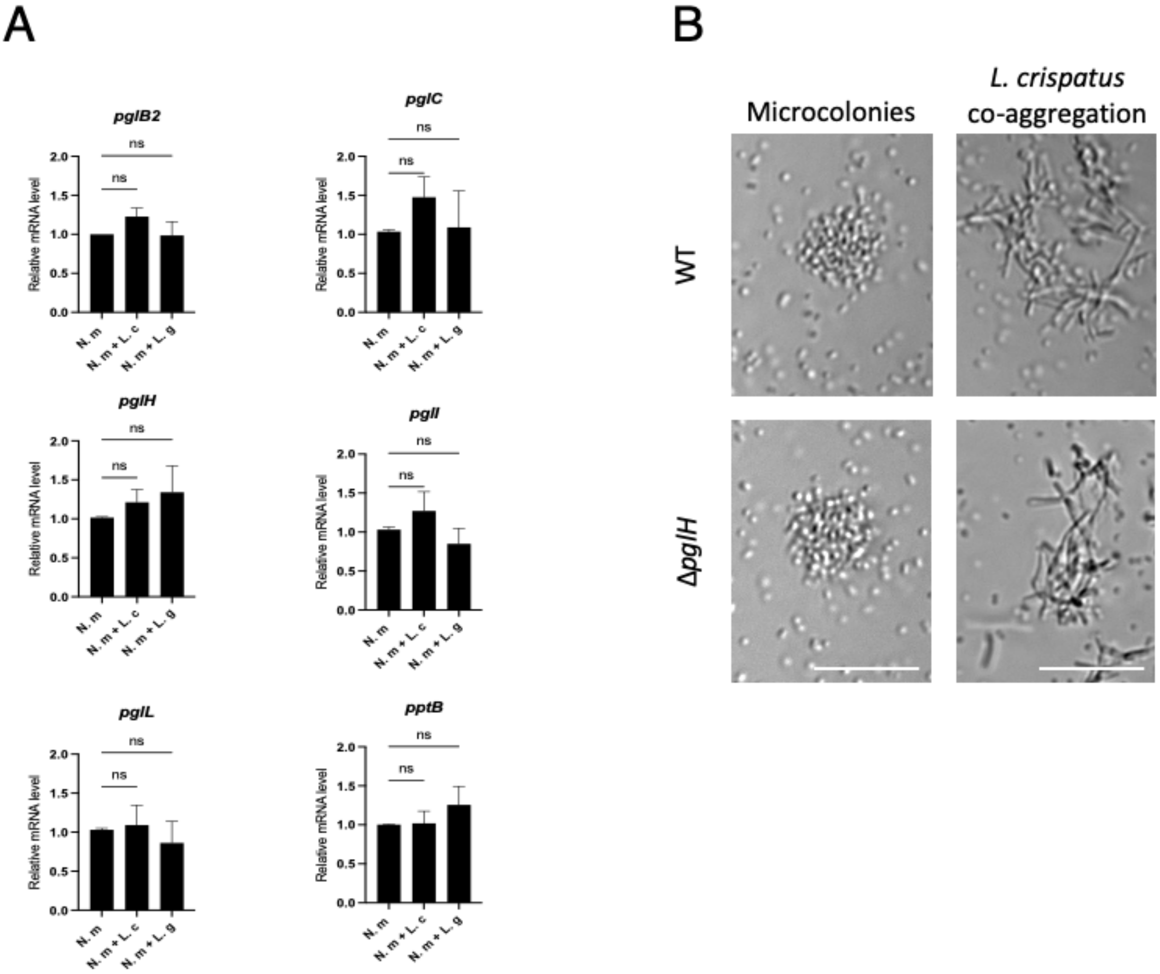

Fig. S4 ***L. cripatus*** **and post translational modification of meningococcal pili**

(A) *L. crispatus* MV24 (L. c) and *L. gasseri* MV1 (L. g) were co-incubated with *N. meningitidis* (N. m) in DMEM supplemented with 1% FBS for 2 h. Bacteria were added at a final concentration of 10^7 CFU/ml each with a ratio of 1:1. Gene expression was determined by qPCR. Data represent the mean ± SD of two independent experiments in triplicates and are presented as the fold change relative to N. m without lactobacilli. Non-significant results are written as ns. (B) Microscopy images of *N. meningitidis* (WT) and N. *meningitidis* Δ*pglH* showing microcolony formation (left images), and co-aggregation with *L. crispatus* MV24 (right images) at 2 h post-incubation. Bacteria were imaged under an Axiovert Z1 Zeiss microscope at 40x magnification. Scale bar, 20 µm.

FIGURE S5

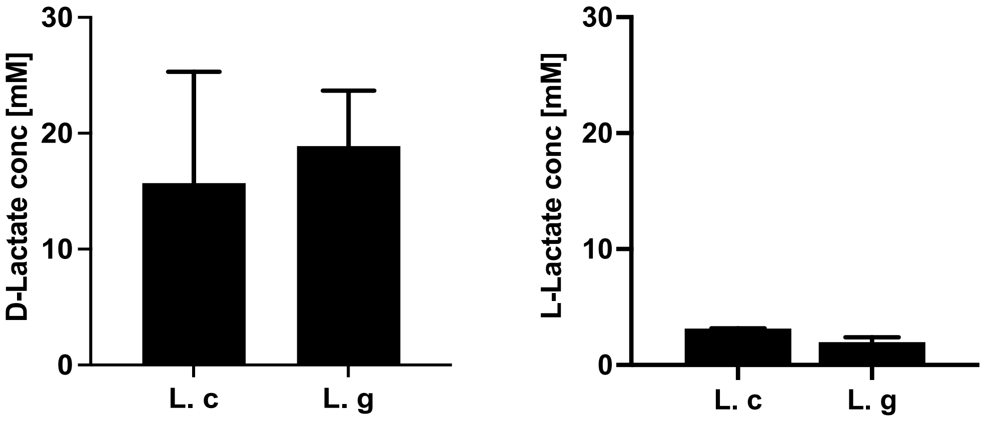

Figure S5. ***L. crispatus*** **and** ***L. gasseri*** **produce both L- and D-lactate.**

L. crispatus (L. c) and *L. gasseri* (L. g) were incubated in DMEM with 1% FBS for 4 h to produce lactate before being sterile-filtered through a 0.2 µm pore filter. Lactate in the supernatant was detected using a lactate detection kit for L- and D-lactate (Sigma-Aldrich) and read at 565 nm using SpectraMax i3x. Two technical repeats were performed for each lactate isomer.

FIGURE S6

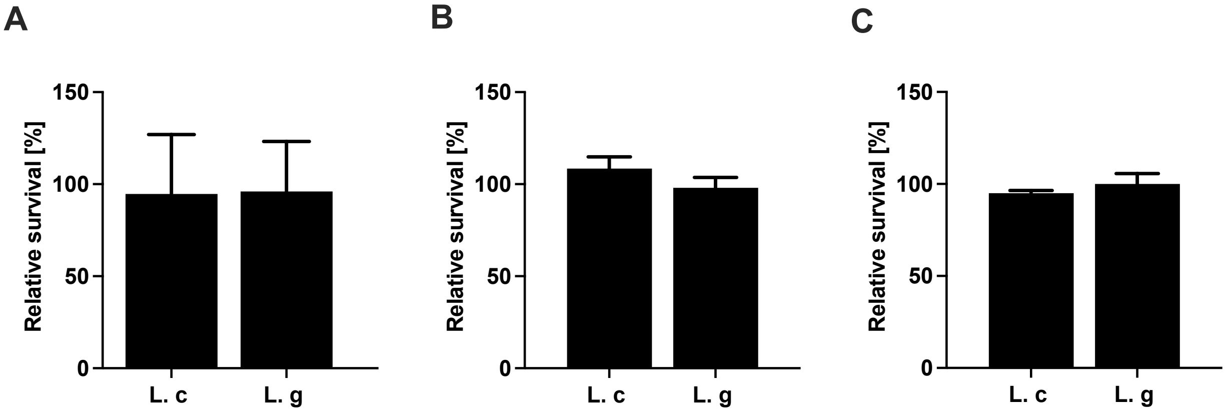

Figure S6*: L. crispatus* and *L. gasseri* were not killed by antimicrobial peptides LL-37, hBD2, or antibiotic cephalexin.

L. crispatus MV24 (L. c) or *L. gasseri* MV1 (L. g) was incubated for 3 h with either a H_2O control or (A) 5 µM LL-37, (B) 5 µM hBD2, or (C) 0.5 µg/µl cephalexin. The quantitative data were obtained by plating for viability on Rogosa agar. Two technical repeats were performed for each of the quantitative experiments.

Table S1. Bacterial strains used in this study

Species	Strain	Reference
Lactobacillus crispatus	MV24	[1, 2]
- ” -	ATCC33820	(1, 2)
- ” -	ATCC33197	(1, 2)
Lactobacillus gasseri	MV1	[1, 2]
Lacticaseibacillus rhamnosus	Kx151 A1	[1, 2]
Ligilactobacillus salivarius	LMG 9477	[1, 2]
Limosilactobacillus reuteri	ATCC PTA 5289	[1, 2]
Neisseria meningitidis	FAM20, WT	[3]
- ” -	Δ*pilC1*, Δ*pilC2*	[3]
- ” -	Δ*pilE*, Δ*siaD*	[4]
- ” -	Δ*pilT*	[5]
- ” -	Δn*afA*	[6]
- ” -	Δ*lpxA*	[7]
- ” -	Δ*pptB*	[8]
- ” -	Δ*pglH*	[9]
N. meningitidis	JB515	[3]
- ” -	Z6466, Z2461,	[10]
- ” -	M96255789	[11]
- ” -	ROU	[12]
- ” -	MC58	[13]

Table S2 Primers used in this study

Primer	Sequence (5'-3')	Reference
*rpsJ*_fwd	TTGGAAATCCGCACCCACTT	[6]
*rpsJ*_rev	TACATCAACACCGGCCGACAAA	[6]
*pglB2*_fwd	ATTTTCAATCTGGCGGTACG	[14]
*pglB2*_rev	AAATTTCGGGTGAGCGTATG	[14]
*pglC*_fwd	GCGATTATCGTGGTTCACCT	[14]
*pglC*_rev	ACCGGTGGTCATGATTTTGT	[14]
*pglH*_fwd	TGCAGTCGGTTACCAACAAA	[14]
*pglH*_rev	TTATCGGCTTGAACGAAACC	[14]
*pglI*_fwd	GAAGAACACCTGCCCCTGTA	[14]
*pglI*_rev	CCATCAGGTAAACGGCTTGT	[14]
*pglL*_fwd	GGCCTGATTGTCCTGTTGTT	[14]
*pglL*_rev	GGGTAACGATGCGTTCTTGT	[14]
*pptB*_fwd	AAGGCGTGGAAGTCATCATC	[14]
*pptB*_rev	TGTTTGAGGTAGGTAGCGGAAGGT	[14]

REFERENCES

1. Roos S, Engstrand L, Jonsson H. Lactobacillus gastricus sp. nov., Lactobacillus antri sp. nov., Lactobacillus kalixensis sp. nov. and Lactobacillus ultunensis sp. nov., isolated from human stomach mucosa. Int J Syst Evol Microbiol. 2005;55(Pt 1):77-82; doi: 10.1099/ijs.0.63083-0.
2. de Klerk N, Maudsdotter L, Gebreegziabher H, Saroj SD, Eriksson B, Eriksson OS, et al. Lactobacilli Reduce Helicobacter pylori Attachment to Host Gastric Epithelial Cells by Inhibiting Adhesion Gene Expression. Infect Immun. 2016;84(5):1526-35; doi: 10.1128/IAI.00163-16.
3. Rahman M, Kallstrom H, Normark S, Jonsson AB. PilC of pathogenic Neisseria is associated with the bacterial cell surface. Molecular microbiology. 1997;25(1):11-25.
4. Engman J, Negrea A, Sigurlásdóttir S, Geörg M, Eriksson J, Eriksson OS, et al. Neisseria meningitidis Polynucleotide Phosphorylase Affects Aggregation, Adhesion, and Virulence. Infect Immun. 2016;84(5):1501-13; doi: 10.1128/IAI.01463-15.
5. Jones A, Georg M, Maudsdotter L, Jonsson AB. Endotoxin, capsule, and bacterial attachment contribute to Neisseria meningitidis resistance to the human antimicrobial peptide LL-37. Journal of bacteriology. 2009;191(12):3861-8; doi: JB.01313-08 [pii] 10.1128/JB.01313-08.
6. Kuwae A, Sjolinder H, Eriksson J, Eriksson S, Chen Y, Jonsson AB. NafA negatively controls Neisseria meningitidis piliation. PLoS ONE. 2011;6(7):e21749; doi: 10.1371/journal.pone.0021749.
7. Albiger B, Johansson L, Jonsson AB. Lipooligosaccharide-deficient Neisseria meningitidis shows altered pilus-associated characteristics. Infect Immun. 2003;71(1):155-62.
8. Sigurlasdottir S, Lidberg K, Zuo F, Newcombe J, McFadden J, Jonsson AB. Lactate-Induced Dispersal of Neisseria meningitidis Microcolonies Is Mediated by Changes in Cell Density and Pilus Retraction and Is Influenced by Temperature Change. Infect Immun. 2021;89(10):e0029621; doi: 10.1128/IAI.00296-21.
9. Gault J, Ferber M, Machata S, Imhaus AF, Malosse C, Charles-Orszag A, et al. Neisseria meningitidis Type IV Pili Composed of Sequence Invariable Pilins Are Masked by Multisite Glycosylation. PLoS Pathog. 2015;11(9):e1005162; doi: 10.1371/journal.ppat.1005162.
10. Davidsen T, Amundsen EK, Rødland EA, Tønjum T. DNA repair profiles of disease-associated isolates of Neisseria meningitidis. FEMS Immunol Med Microbiol. 2007;49(2):243-51; doi: 10.1111/j.1574-695X.2006.00195.x.
11. Johnson CR, Newcombe J, Thorne S, Borde HA, Eales-Reynolds LJ, Gorringe AR, et al. Generation and characterization of a PhoP homologue mutant of Neisseria meningitidis. Mol Microbiol. 2001;39(5):1345-55; doi: 10.1111/j.1365-2958.2001.02324.x.
12. Klee SR, Nassif X, Kusecek B, Merker P, Beretti JL, Achtman M, et al. Molecular and biological analysis of eight genetic islands that distinguish Neisseria meningitidis from the closely related pathogen Neisseria gonorrhoeae. Infect Immun. 2000;68(4):2082-95; doi: 10.1128/iai.68.4.2082-2095.2000.
13. Tettelin H, Saunders NJ, Heidelberg J, Jeffries AC, Nelson KE, Eisen JA, et al. Complete genome sequence of Neisseria meningitidis serogroup B strain MC58. Science (New York, NY. 2000;287(5459):1809-15; doi: 10.1126/science.287.5459.1809.
14. Sigurlasdottir S, Engman J, Eriksson OS, Saroj SD, Zguna N, Lloris-Garcera P, et al. Host cell-derived lactate functions as an effector molecule in Neisseria meningitidis microcolony dispersal. PLoS Pathog. 2017;13(4):e1006251; doi: 10.1371/journal.ppat.1006251.
